# Tobacco use and its associated factors among students of medical college at tertiary care center of Eastern Nepal

**DOI:** 10.1371/journal.pone.0296592

**Published:** 2024-07-31

**Authors:** Pratik Adhikari, Pramodman Singh Yadav, Rama Khadka, Sujan Kafle, Anusha Rayamajhi, Min Raj Bhurtel, Santosh Adhikari, Manisha Shrestha, Varsha Chettri, Manish Upreti, Prajwal Gautam, Shreya Dhungana, Suyash Dawadi, Prakriti Adhikari, Aakash Koirala

**Affiliations:** 1 B.P. Koirala Institute of Health Sciences, Dharan, Nepal; 2 Kathmandu University School of Medical Sciences, Dhulikhel, Nepal; 3 Chitwan Medical College, Bharatpur, Nepal; Shahid Beheshti University of Medical Sciences School of Dentistry, ISLAMIC REPUBLIC OF IRAN

## Abstract

**Background:**

Despite declining smoking prevalence globally, South Asia faces a rising burden. In Nepal, existing tobacco control laws haven’t curbed use, with 28.9% of young adults engaging in tobacco use. This study investigates tobacco use and associated factors among medical, dental, and nursing students at a Nepalese tertiary care center.

**Objective:**

We aimed to assess tobacco use prevalence and identify factors associated with it among future healthcare professionals, considering their distinct roles in tobacco control. Medical students can contribute through clinical counseling and public health advocacy, dental students through oral health education, and nursing students through patient education and community outreach.

**Methods:**

A cross-sectional study involving 427 medical, dental, and nursing students was conducted. Data was collected using online questionnaires distributed via email and social media. Descriptive statistics and Chi-square tests were used for analysis.

**Results:**

The study found that 45% of participants were aged 22–25, with females comprising the majority (53.2%). Nearly half (49.2%) belonged to the medical faculty, and 24.4% were in their first year. Furthermore, among those who smoke, 53% reported smoking less than 5 cigarettes daily. The analysis revealed significant associations between smoking with age (p = 0.01), year of study (p = 0.001), parental smoking history (p = 0.001), and having friends who smoke (p = 0.001).

**Conclusion:**

Our findings highlight the moderate prevalence of cigarette smoking among medical students, with family and friends emerging as major influences. Stress relief was a common reason, particularly among young females and first-year students. These results emphasize the urgent need for comprehensive tobacco control programs within medical institutions to equip future healthcare professionals to effectively address smoking issues.

## Introduction

Smoking is demonstrably detrimental to health, linked to numerous health risks, premature death, and severe morbidity [1–4]. Unfortunately, while developed countries see a consistent decline in smoking prevalence, it’s on the rise in many developing nations, including Nepal [5]. Global cigarette production outpaces population growth, and the World Health Organization (WHO) predicts 10 million annual deaths attributable to tobacco use by 2030 [5, 6]. Due to these significant public health consequences, the WHO prioritizes tobacco control initiatives [6].

In South Asia, an estimated 1.2 million people die annually from tobacco-related causes. Despite tobacco control laws in Bangladesh, Nepal, and Sri Lanka, youth tobacco use remains widespread and concerning [7]. The 2019 WHO STEPwise Approach to NCD Risk Factor Surveillance (WHO-STEPS) survey in Nepal revealed that 28.9% of individuals aged 15–69 currently use smoked or smokeless tobacco products. These numbers haven’t shown a significant decrease since 2013, despite implemented control laws [8].

Approximately 70% of early adult deaths are linked to behavioral patterns established in adolescence, including smoking [9]. The undergraduate years, marking the transition from adolescence to young adulthood, are a time of increasing independence and potential risk-taking behaviors [9–11]. Studies have shown that adolescents who are offered free tobacco products are more likely to smoke [11].

Healthcare professionals play a vital role in discouraging tobacco use by educating their patients about its dangers and serving as role models for the public [12]. Their participation in prevention and cessation counseling is crucial for reducing tobacco-related deaths. However, research suggests that smoking healthcare professionals are less effective in convincing patients to quit [13, 14]. Understanding healthcare professionals’ own tobacco use patterns is therefore essential before implementing anti-tobacco measures. A recent study among public health students in Kathmandu found a notable prevalence of tobacco use, with nearly 40% having ever smoked and over 16% being current smokers. This study also identified factors associated with smoking, such as male gender, older age, ethnicity, living arrangements, parental smoking history, and friends who smoke [15].

This study, conducted among undergraduate medical students at the Bishweshwar Prasad Koirala Institute of Health Sciences, aimed to assess the prevalence and associated factors of tobacco use, secondhand smoke exposure, motivations for smoking, cigarette access, and tobacco cessation behaviors. Similarly, the current research investigates tobacco use and related factors among medical, dental, and nursing students at a tertiary care center in Eastern Nepal. Recognizing the unique roles of each discipline–clinical counseling and public health advocacy, and other relevant activities, oral health education, and screenings, and patient education and community outreach–this study seeks to understand how medical students, for example, can contribute to effective tobacco control efforts. Dental students can focus on oral health education and screenings, leveraging their expertise in dental care. Nurses, on the other hand, can provide patient education and engage in other relevant activities within community outreach programs, utilizing their position to disseminate information and promote healthy behaviors.

## Materials and methods

This research employs a descriptive cross-sectional design to examine tobacco use prevalence among undergraduate medical students at the Bishweshwar Prasad Koirala Institute of Health Sciences (BP Koirala Institute) in Dharan, Nepal. The study focuses specifically on students enrolled in the Bachelor of Medicine, Bachelor of Surgery (M.B.B.S.) and Bachelor of Dental Surgery (B.D.S.) programs.

### Study population and sample size

The study population encompasses all full-time undergraduate medical students at BP Koirala Institute. A sample size of 427 was determined based on a 95% confidence interval and a 5% margin of error. According to the STEPS Survey Nepal, 2019, the expected prevalence of tobacco use in the population was taken as 29% [8]. The sample size was calculated using Cochran’s formula.

Using the formula,

n = Z^2^Pq/d^2^

- n = sample size
- Z = standard normal deviate at the desired confidence level (1.96 for 95% confidence)
- p = estimated prevalence of tobacco use (assumed to be 27.1% based on a related study)
- q = 1—p (represents the proportion of the population not using tobacco)
- d = margin of error (set at 0.05)
- n = (1.96)^2^ × 0.29 × 0.73/(0.05)^2^

N = 325

Therefore, the calculated sample size was 325, and considering 23.84% nonresponse rate, the sample size obtained was 427. The study was conducted from October 5th to December 5th, 2023. Invitations to participate were sent to 500 students out of a total population of 600. Ultimately, 427 students participated, and 77 declined. A systematic random sampling technique was employed to reduce selection bias and improve representativeness.

### Operational definitions

Key study concepts were defined operationally to ensure clarity and consistency in understanding and measurement. These definitions include:

- **Tobacco Use Categories:** Smoking and smokeless tobacco use
- **Prevalence of Tobacco Use:** The proportion of participants who reported current or past use of tobacco products
- **Associated Factors:** Sociodemographic factors (age, gender, course year, faculty, parental smoking history, friends with smoking behavior), motivations for tobacco use, secondhand smoke exposure, and tobacco contacts
- **Behavior Related to Tobacco Cessation:** Quit attempts and successful tobacco cessation

### Measurements

The study utilized the Global Health Professions Student Survey (GHPSS) questionnaire, developed collaboratively by the World Health Organization (WHO), the US Centers for Disease Control and Prevention (CDC), and the Canadian Public Health Association [16]. According to the survey, a "current smoker" is defined as someone who smoked cigarettes daily or occasionally during the 30 days prior to the survey. An "ex-smoker" refers to an individual who previously smoked tobacco but has since quit, excluding current smokers and those who have never smoked. A "nonsmoker" denotes someone who has never smoked a cigarette in their lifetime. Additionally, the survey identifies other tobacco products, such as chewing tobacco, snuff, bidis, hookah, cigars, or pipes [16].

The GHPSS questionnaire was translated into Nepali using a standardized forward-backward translation process to ensure linguistic and cultural adaptation. This process involved translating the English version into Nepali by a bilingual expert, followed by back-translation into English by a separate bilingual expert to verify the accuracy and equivalence of the translation. Any discrepancies were resolved through consensus.

A pilot test of the translated questionnaire was conducted with a small group of 30 undergraduate medical students at BP Koirala Institute to ensure clarity, relevance, and comprehensibility in the local context. Feedback from the pilot test was used to make minor adjustments to the questionnaire. The pilot testing also served to validate the questionnaire’s reliability and validity within the local context.

To mitigate potential social desirability bias in self-reported smoking behavior, anonymity and confidentiality of responses were assured. Participants were informed that their responses would remain confidential and would only be used for research purposes. Additionally, the questionnaire was self-administered online, reducing the likelihood of interviewer bias and allowing participants to complete the survey in a private setting.

### Data collection

Data collection involved the administration of an online validated questionnaire. A self-administered validated GlobalHealth Professions Student Survey (GHPSS) questionnaire, a validated tool for screening smoking among university students [16], was used for this study, which was developed by the World Health Organization (WHO), the US Centers for Disease Control and Prevention (CDC), and the Canadian Public Health Association [17]. The questionnaire was distributed via email and social media platforms to undergraduate medical students at BP Koirala Institute using Google Forms. Google Forms settings were configured to ensure a single response from each student ("Limit to 1 response"). Data collection was facilitated by medical officers and physicians working in the Department of Internal Medicine at the institute. This method offered a convenient means for student participation while promoting accurate and reliable data collection.

### Data analysis

Collected data was entered into Microsoft Excel 2010 and then transferred to SPSS version 11.5 for statistical analysis. Descriptive statistics, including frequency, percentage, mean, standard deviation, and median, were employed to characterize sociodemographic variables. Pearson’s correlation coefficient and Chi-square tests were used as appropriate to examine associations between tobacco use and selected sociodemographic variables (age, gender, course year, faculty, parental smoking history, and friends with smoking behavior). The level of significance was set at p < 0.05 throughout the study. The number of participants’ responses included in the discrete statistical analyses varied due to missing data for certain variables. This systematic approach to data analysis aims to provide a comprehensive overview of tobacco use patterns and associated factors among the targeted population of medical students at BP Koirala Institute in Dharan.

## Results

This section presents the key findings of a study investigating moderate prevalence of tobacco use among undergraduate medical students at B.P. Koirala Institute of Health Sciences (BPKIHS) in Dharan, Nepal. Data are summarized in tables, with detailed information provided for further reference.

### Sociodemographic characteristics

Table 1 depicts the demographic profile of the 427 respondents indicates a majority of females (227, 53.1%) compared to males (200, 46.9%). The most prevalent age group among participants was 22–25 years (192, 44.9%), followed closely by individuals aged ≤ 21 years (173, 40.5%) and those older than 25 years (62, 14.5%). Academic year distribution showed a relatively even spread, with 1st-year students numbering 104 (24.4%), 2nd-year students at 83 (19.4%), 3rd-year students at 72 (16.9%), 4th-year students at 66 (15.5%), and 5th-year students at 102 (23.9%). Among the disciplines, the College of Medicine boasted the highest representation (210, 49.2%), followed by Dentistry (111, 26%) and Nursing (106, 24.8%).

**Table 1:** Socio-demographic characteristics of the respondents. n = 427.

Characteristic	Category	Number of Respondents	Percentage
Age in years	≤ 21 years	173	40.5
	**22–25 years**	**192**	**45.0**
	>25 years	62	14.5
Gender	Male	200	46.8
	**Female**	**227**	**53.2**
Course Year	**1**^**st**^ **year**	**104**	**24.4**
	2^nd^ year	83	19.4
	3^rd^ year	72	16.9
	4^th^ year	66	15.5
	5^th^ year	102	23.9
Faculty	**Medicine**	**210**	**49.2**
	Dentistry	111	26.0
	Nursing	106	24.8

### Tobacco use prevalence

**Fig 1:**
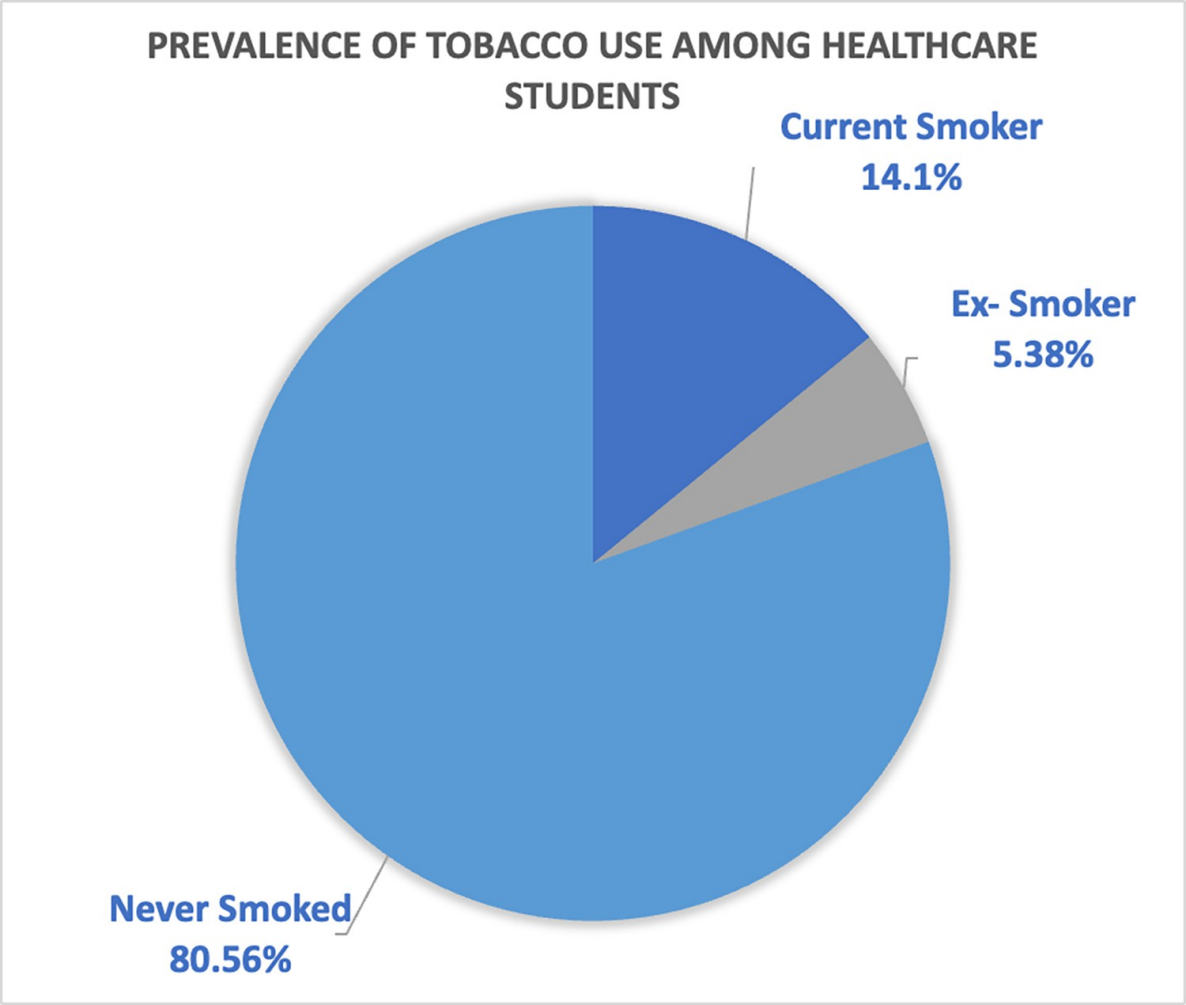
Moderate prevalence of tobacco use among healthcare students (n = 427). Current Smoker (14.1%)Ex-Smoker (5.38%)Never Smoked (80.56%).

**Fig 1** depicts the moderate prevalence of tobacco use among participants. The pie chart shows that exactly 14.1% (n = 60) were current smokers, while 5.4% (n = 23) reported a history of smoking (ex-smokers). The remaining 80.56% (n = 344) had never smoked.

### Smoking characteristics (current and ex-smokers)

**Table 2** delves deeper into smoking characteristics among current smokers (n = 60). It reveals that a notable portion (around 41%) had been smoking for a moderate duration (1–4 years). Daily cigarette consumption was the most common behavior, followed by smoking a moderate number of cigarettes (5–10) per day. Stress relief and enjoyment/pastime emerged as the primary reasons for smoking among these participants.

**Table 2:** Smoking characteristics among smokers (current and ex-smokers) (n = 83). n = 83.

Items	Category	Number of Respondents	Percentage
Smoking Duration	< 1 years	20	24
	**1–4 years**	**34**	**41**
	>4 years	29	35
Cigarettes/day	**< 5 / day**	**44**	**53**
	5–10 / day	23	28
	>10 / day	16	19
Reasons behind smoking	**For stress relief**	**34**	**41**
	For attraction	14	17
	For fun / pass time	32	39
	Others	3	4

### Exposure to environmental tobacco smoke

**Table 3** summarizes data on exposure to environmental tobacco smoke. A significant portion of participants reported exposure at home (n = 130, 30.4%) and with friends (n = 186, 43.6%). Daily exposure with friends (who smoke) while smoking was the most frequent category (n = 52, 27.9%).

**Table 3:** Exposure to environmental tobacco smoke (n = 427).

Items	Category	Frequency	Percentage
Is there any smoker in your family?	**Yes**	**130**	**30.4**
	No	297	69.6
Do you sit with family members while smoking?	**Yes**	**75**	**17.6**
	No	352	82.4
Regarding your friends, do you usually sit with smokers while smoking?	**Yes**	**186**	**43.6**
	No	241	56.4
If yes, how many times do you sit with friends(smokers) while smoking.	Daily	52	27.96
	3 to 4 days per week	27	14.5
	Weekends	37	19.9
	**Occasionally**	**70**	**37.6**

### Smoking cessation behavior (current smokers only)

**Table 4** highlights smoking cessation behavior among current smokers (n = 60). A significant majority (n = 45, 75.0%) had attempted to quit smoking in the past. Moreover, a substantial majority (n = 42, 70.0%) expressed an intention to quit smoking at the present time.

**Table 4:** Smoking cessation behavior among current smokers (n = 60).

Items	Category	Frequency	Percentage
**Did you try to quit smoking cigarettes in the past?**	**Yes**	**45**	**75**
	No	15	25
**Do you plan to quit smoking cigarettes now?**	**Yes**	**42**	**70**
	No	18	30

### Association of smoking with independent variables

**Table 5** presents the association of smoking with independent variables. The association between categorical independent variables and categorical dependent variables was measured by the Pearson Chi-square test followed by binary logistic regression analyses.

**Table 5:** Association of smoking with independent variables (n = 427) according to the Pearson chi-square test followed by binary logistic regression analysis.

Characteristics	Categories	Smoking status		P-value	Unadjusted Odds ratio (95% CI)
		Current smoker	Not current smoker		
**Age in years**	**25**	**45**	**320**	**0.01**	**1.4 (1.1–1.8)**
	**>25**	**15**	**47**		
**Gender**	**Male**	**46**	**154**	**0.001**	**2.8 (1.3–5.8)**
	**Female**	**14**	**213**		
**Course year**	**3**^**rd**^ **years**	**22**	**236**	**0.001**	**1.3 (1.1–1.5)**
	**>3**^**rd**^ **years**	**38**	**131**		
**Faculty**	**Medical (MBBS/BDS)**	**45**	**276**	**1**	**0.8 (0.4–2.1)**
	**Paramedical (Nursing)**	**15**	**91**		
**Parental history of smoking**	**Yes**	**41**	**89**	**0.001**	**6.3 (3.2–12.2)**
	**No**	**20**	**277**		
**Friends with smoking behavior**	**Yes**	**56**	**130**	**0.001**	**19.3 (6.4–58.3)**
	**No**	**4**	**237**		

A statistically significant association (p < 0.05) was observed between smoking and age, gender, course year, parental smoking history, and having friends with smoking behavior.

There was a significant association between the age of the participants and smoking status. Lower age groups (unadjusted OR: 1.4, 95% CI: 1.1–1.8) were more likely to be current smokers than higher age groups. Male participants were (unadjusted OR: 2.8, 95% CI: 1.3–5.8) times more likely to be current smokers as com- pared to females. Participants who are in less than or equal to 3rd year (unadjusted OR: 1.3, 95% CI: 1.1–1.5-) were more likely to be current smokers than participants who are in more than 3rd year. Medical (MBBS/BDS) participants (unadjusted OR: 0.99, 95% CI: 0.8–1.2) were less likely to be current smokers as paramedical (Nursing) participants. Compared with participants with a ’No’ response to parental history of smoking, participants with ’Yes’ response to parental history of smoking (unadjusted OR: 6.3, 95% CI: 3.2–12.2) were more likely to be current smokers. Participants who have friends with smoking behavior (unadjusted OR: 19.3, 95% CI: 6.4–58.3) had more odds of being current smokers than those who did not have friends who used tobacco.

### Non-significant finding

It is important to note that no statistically significant association was found between the faculty a student is enrolled in (Medicine, Dentistry, or Nursing) and their smoking status (p = 0.97).

## Discussion

This study investigated the prevalence and characteristics of tobacco use among healthcare student populations at BP Koirala Institute of Health Sciences (BPKIHS) in Dharan, Nepal. Our findings revealed a moderate prevalence of cigarette smoking, with 14.1% of participants identified as current smokers and 5.4% reporting a history of smoking. Notably, the majority (85.9%) of ex-smokers were female students.

While our study shows a lower prevalence compared to the 33% reported among medical students at King Saud University (KSU) in Saudi Arabia [18], it remains higher than the median (13.5%) observed in previous studies conducted among healthcare students and university students in Saudi Arabia over the past two decades [19–23]. These variations highlight the influence of sociocultural factors and tobacco control policies across different regions.

Our findings suggest that a proportion of participants-initiated smoking during adolescence or a young age. This aligns with existing literature, which demonstrates a higher risk of continued smoking and lower likelihood of cessation for individuals who start smoking young [18]. This trend raises concerns about the potential long-term health consequences for these students, who may become future healthcare providers.

This study found no significant association between smoking and the faculty a student belonged to (Medicine, Dentistry, or Nursing). Sychareun et al. [24] findings align with this, suggesting that factors beyond academic discipline may influence smoking behavior.

Stress relief emerged as the primary reason for smoking among participants, mirroring findings from studies on medical students in Northern Ethiopia and Saudi Arabia [25, 26]. Other reported reasons included enjoyment and social attraction.

A significant proportion of participants reported exposure to second-hand smoke, both at home (17.6%) and with friends (43.6%). This aligns with a meta-analysis by Fischer et al. which supports the association between second-hand smoke exposure and various health problems [27].

While not entirely groundbreaking compared to all Nepali research, this study offers valuable insights within the specific context of healthcare student populations. Previous studies in Nepal have explored tobacco use prevalence among university students and medical students in general, but this investigation focuses specifically on healthcare students at BPKIHS. This targeted approach allows for a more nuanced understanding of smoking behavior within a future healthcare workforce.

Combined with the findings on second-hand smoke exposure, this study highlights the need for interventions to reduce tobacco use and exposure among healthcare students. Campus-wide anti-smoking policies and awareness campaigns could be implemented. These programs could also explore ways to mobilize healthcare students as advocates for tobacco control in their own communities.

### Limitations

This study has inherent limitations. The cross-sectional design provides a single point in time snapshot, hindering the establishment of cause-and-effect relationships between tobacco use and other factors. Additionally, while recruitment was limited to a single medical school, Bishweshwar Prasad Koirala Institute of Health Sciences (BP Koirala Institute) in Eastern Nepal, this focus allowed for in-depth exploration of tobacco use patterns specifically among undergraduate medical and dental students. The findings provide valuable insights into the prevalence and correlates of tobacco use within this specific academic setting.

Self-reported data is another limitation, which can be susceptible to recall bias. Furthermore, important confounding factors like socioeconomic status, stress levels, and access to smoking cessation resources were not fully explored. Consequently, definitive conclusions regarding the prevalence and causes of tobacco use among healthcare students cannot be drawn from this study. However, the study’s comprehensive approach to data collection and analysis contributes to understanding tobacco use behaviors within this specific population.

The exclusive focus on smoked tobacco products limits the scope to smoking behaviors among healthcare students. Future research employing longitudinal designs and including a broader sample from various healthcare institutions in Nepal could provide more robust insights. Investigating the underlying reasons for smoking initiation and cessation attempts among healthcare students could also be valuable. Additionally, considering the study’s exclusive focus on smoked tobacco products, future investigations should explore smokeless tobacco products among healthcare students to offer a more comprehensive understanding of tobacco consumption in this demographic.

## Conclusions

Cigarette smoking is highly prevalent among both male and female medical students in the Faculty of Medicine, Dentistry, and Nursing. Contacts (family and friends) who smoke are the major risk factors for initiating the behavior. Regarding the reason behind smoking, nearly half of the students have answered smoking for stress relief, followed by 38.6% for fun/time pass, 16.9% for attraction, and the remaining 3.6% as other reasons. Based on the results of the findings, the study concluded that nearly half of the respondents fall between the ages of 22–25%, more than half of the respondents are female, nearly half are from the medicine first faculty, nearly a quarter of the respondents are from the first year, and nearly half have responded that they do like smoking because smoking has negative effects on health. Among smokers, nearly more than half smoke <5 cigarettes per day. Furthermore, the study also illustrates that there was a significant association between smoking and age in years, course year, parental history of smoking, and friends with smoking behavior.

All possible efforts and strategies should be considered to curb smoking among medical students in Nepal and globally, which should encompass a comprehensive approach. Educational institutions, particularly medical and health colleges, must integrate dedicated programs and courses on tobacco control. This initiative aims to empower future healthcare professionals with the requisite knowledge and skills to effectively address smoking issues.
